# Annotations

**Published:** 1901-01-19

**Authors:** 


### Annotations.

### The Municipalisation of Hospitals.

While so many busybodies are calling out for the municipalisation of the hospitals in London it is not uninstructive to do what we are so often told to do by our self-appointed mentors, namely to look to the United States and see what is going on in this respect in a really free and enlightened community. They evidently are not quite happy about their hospital arrangements in New York. At their great municipal hospital, the Bellevue, which is managed by the commissioners of charities, many abuses have lately been brought to light, especially in regard to the brutality of the male nurses in the alcoholic wards and in the lunatic department, three of the attendants in which are now charged with the murder of a lunatic under their care. The whole system of management of this institution has recently come under review, and the blame of the condition of affairs which has been discovered is being largely thrown on the system of municipal management which is in force. Whatever else may be the outcome a reorganisation of the entire system of municipal management of public hospitals seems to be inevitable. The management of Bellevue is, we are told, thoroughly political in character from top to bottom. It is complained that it is impossible to maintain real discipline among employés whose places are made for them by political "bosses," and it is shown that it may not be an easy thing to discharge an incompetent superintendent, a brutal nurse, or a drunken ambulance driver, who may be the friend of a "ward leader." Under all these circumstances we can hardly wonder that the cry is raised for an amendment of the charter in such a form as to give the hospitals into the charge of an independent board of citizens. In London, on the other hand, certain doctrinaires are so smitten with the beauties of municipalisation that they are all for extending that somewhat doubtful blessing even to our hospitals, and for upsetting what the Americans are so greatly longing for—namely, boards of management entirely independent of political influences.

### Yellow Fever.

The question of the origin of yellow fever, which has for long exercised the minds of American physicians, has become a matter of still greater interest since the acquisition of Cuba by the United States. In former days the States had to remain on the defensive every summer to guard against the introduction of the disease, and they held Cuba to blame as the origin and starting point of most of their trouble in this regard. Now, however, that Cuba has become an American possession, they can no longer find a scapegoat. They are now themselves responsible for the condition of Havanna and other yellow fever ports, and they have set manfully to work to discover the root of the evil. There were two theories on the question. One party held that yellow fever was produced by the *bacillus icteroides*, discovered by Sanarelli, while the other, regarding the disease as the result of inoculation by mosquito bites, looked for some blood condition analogous to that by which malaria is produced. Oddly enough, while the naval service officially backed the one theory, the Army Medical Service supported the other. Now, however, it is announced that the mosquito theory has definitely won the day. It may be as well, however, not to be too hasty; we are always a little suspicious about medical discoveries which are announced by telegraph. The experiments seem to have taken two directions. In one set it was sought to give protection against the fever by gradual inoculation by means of infected mosquitoes, much on the lines of the experiments undertaken years ago by Dr. Finlay, and it is said that these have now proved successful, that is, that the inoculation has "taken" in a large proportion of the cases. The experiments were performed on immigrants who had not had the disease, and the fever produced is said to have been typical. On the other hand a different set of researches have been in progress. Soldiers are, it is said, exposing themselves to what, according to the older theory, would be deadly risks by sleeping in the clothes used by infected patients, but are all the while taking care to protect themselves from the bites of mosquitoes. So far the results are said to be favourable, the soldiers having escaped infection; all of which points strongly to the probability of the theory that the disease is transmitted by mosquitoes rather than by the more generally recognised modes of infection.

### The Provision for Inebriates.

The first report of the inspector of certified reformatories under the Inebriates Act, 1898, has just been issued by the Home Office, and although it cannot be said that it shows a very satisfactory condition of affairs it does at least give indications that something is being done to pave the way for a fuller application of the Act than has hitherto been possible. This Act contemplates the establishment of two distinct types of institution—namely, State and certified reformatories—and the committal of two distinct classes of inebriates, those, namely, who are convicted of indictable offences committed under the influence of drink, or attributable to it, and those who may be called "habitual drunkards," as shown by their having been repeatedly convicted of drunkenness and disorderly conduct. According to the observations made by the inspector, Mr. Branthwaite, both these classes are equally amenable to discipline when once the liquor has been removed, and he is of opinion that the State inebriate reformatory of the future should not be designed specially for the reception of cases under section 1, but should be adapted for the reception of all intractable cases under either section. It is somewhat disappointing to find that during the first year of the operation of the Act only five certificates for reformatories were applied for; but, on the other hand, it is satisfactory to hear that the magistrates throughout the country, appear as a whole, anxious to take full advantage of the Act as soon as reformatories are available. A very important matter is brought forward in regard to the treatment of habitual inebriates, which it would be well always to bear in mind even in ordinary practice, and that is the necessity of weaning the drinker from the habit of mere drinking, quite irrespective of drinking alcohol. The inebriate becomes a thirsty soul, and if during his reformation he is allowed to drink indiscriminately, even of non-alcoholic fluids, he is much more exposed to danger of relapse than if before discharge he is broken of what one may call the "habit of thirst"; for all the tissues of the inebriate have become so habituated to excess of fluid *qua* fluid that there is a constant danger that this habit may lead a patient, when his course of treatment is over, to revert almost unconsciously to alcoholic beverages.

